# The Urban Gradient in Malaria-Endemic Municipalities in Acre: Revisiting the Role of Locality

**DOI:** 10.3390/ijerph15061254

**Published:** 2018-06-13

**Authors:** Ana Paula Dal’Asta, Raquel Martins Lana, Silvana Amaral, Cláudia Torres Codeço, Antônio Miguel Vieira Monteiro

**Affiliations:** 1 Divisão de Processamento de Imagens (DPI), Instituto Nacional de Pesquisas Espaciais (INPE), Av. dos Astronautas 1758, São José dos Campos 12227-010, Brazil; silvana@dpi.inpe.br (S.A.); miguel@dpi.inpe.br (A.M.V.M.); 2 Programa de Computação Científica (PROCC), Fiocruz—RJ, Av. Brasil 4365 Residência Oficial, Manguinhos, Rio de Janeiro 21041-222, Brazil; raquelmlana@gmail.com (R.M.L.); claudia.codeco@fiocruz.br (C.T.C.)

**Keywords:** urban gradient, Amazônia, malaria, urban-rural linkages

## Abstract

Urbanization has altered the distribution of diseases of public health importance along gradients of human occupation. Adopting dichotomous urban/rural categories to explain differences in the risk of exposure, as well as the prevention of diseases is insufficient. In this paper, we present the potential of representations based on the gradient perspective to characterize the living spaces of municipalities where malaria is endemic in northwest Acre. Inventoried data in 40 localities in the Mâncio Lima and Rodrigues Alves municipalities and information on land use and land cover obtained from the TerraClass Database were used to characterize the urban spatial forms and their social content. Results showed a gradient of intensities: from municipal seats to the most connected localities through the road network to riverine communities. Based on the results, we hope to advance the discussion about the use of normative definitions of “urban” and “rural” for public policies and actions to control and eliminate malaria, considering the position of each locality in its own locally referenced urban gradient.

## 1. Introduction 

The contemporary organization of everyday life spaces has reshaped urban-rural linkages and blurred the once clear and dichotomous notions of urban and rural spaces. Several studies postulate the idea of a complex and ambiguous landscape formed by gradients of urbanicity and rurality [1,2,3,4]. Considering public health problems, the distinction between urban and rural health is important to understanding the differences in disease burden that are associated with environmental, social, economic, and cultural characteristics that change during the urbanization process [5,6,7]. For example, urbanization has been found to be associated with higher prevalence of directly transmitted diseases, chronic diseases such as type 2 diabetes, hypertension, and metabolic syndromes [8].

Despite the clear association between health and urbanization, when dealing with local settings clear-cut definitions have limitations and are restrictive because the variability of and interrelationships between intra-urban and intra-rural life are not taken into account [8,9,10,11]. In the United States, Hall et al. [12] observed that a dichotomous rural-urban classification masked hidden heterogeneity in very rural areas in their access to health care. The same was observed within urban areas in Kenya [13] and by Cohen et al. [14] who concluded when assessing rural-urban health disparities in older Americans adults, that associations are more complex than simply “rural vs. urban”. Classifying territorial diversity and heterogeneity is important to establish clear and appropriate associations in epidemiological studies [15,16,17], and for allocating interventions in areas where they are most needed [7]. 

The focus of this study is malaria in the Brazilian Amazon. Malaria is a disease caused by multiple *Plasmodium* spp. vectored by *Anopheles* mosquitoes and it is an example of a disease whose epidemiology requires a more complex definition of urbanization. Traditionally associated with poor rural settings, the burden of malaria generally decreases as urbanization increases since landscape transformation tends to reduce the ecological conditions for maintenance of the mosquito vector [18]. However, in several countries of Africa, Asia and Americas, populations that are classified as urban are at risk of malaria. In general, these urban populations are surrounded by malaria-endemic rural areas, and urban malaria cases can occur due to transmission in the outskirts of urban areas, or in green pockets within cities, or in rural areas where the urban population usually go for work or leisure. Meanwhile, within rural areas, malaria is also very heterogeneously distributed. In the Brazilian Amazon, for example, increased risk of malaria is associated with specific rural landscapes, for example, recent settlements located at the forest fringe or associated with activities such as mining and logging [19,20]. Besides, urban-rural linkages, due to the presence of ruralities in the urban areas and population mobility, have also been identified as an important factor in the occurrence of urban malaria in the Amazon [21,22].

The Alto Juruá area, in the west of Brazil, is one of the main hotspots of malaria in the Americas [23]. This hotspot is characterized by persistent endemicity, high spatial heterogeneity, presence of autochthonous transmission in urban areas, high commutation of the population between rural and urban localities, and fast land transformation [24]. All these factors have been shown to induce gradients of malaria incidence among rural and urban communities. Lana et al. [19] carried out a survey in 40 localities within this hotspot, including a range of urban and different types of rural settlements. Using correspondence analysis, they found evidence of association between malaria incidence and household traits associated with increasing urbanization, such as electricity, house construction material, appliances, etc. Their results suggest that a more complex urban-rural taxonomy would be useful for classifying areas with different risks for malaria.

Meanwhile, a method for classifying the urbanization process in the Amazon as a continuum was proposed by Dal’Asta et al. [25]. Based on the theory of extensive urbanization [26], the framework builds upon a model with three dimensions: objects, actions and values. This classification scheme has been proved useful for characterizing urbanization in the Southeast Amazon, in the state of Pará. Urban life has extended beyond the cities to influence rural life, thus contributing to the discussion brought by the geographer Bertha Becker on the need for an urban agenda for the Amazon [27,28]. Becker proposed the term, urbanized forest [29], seeking to create a strong image for the inclusion of urban issues in the geopolitical agenda for the region. 

The objective of the present study is to further apply the urbanization classification method proposed by Dal’Asta et al. [25] to describe gradients of malaria risk within the Alto Juruá malaria hotspot characterized by Lana et al. [19]. Understanding local determinants of malaria along this gradient, in landscapes that are not primarily urban or rural, is essential to advance the development of intervention strategies to minimize the burden of disease.

## 2. Materials and Methods 

### 2.1. Study Area

**Figure 1:**
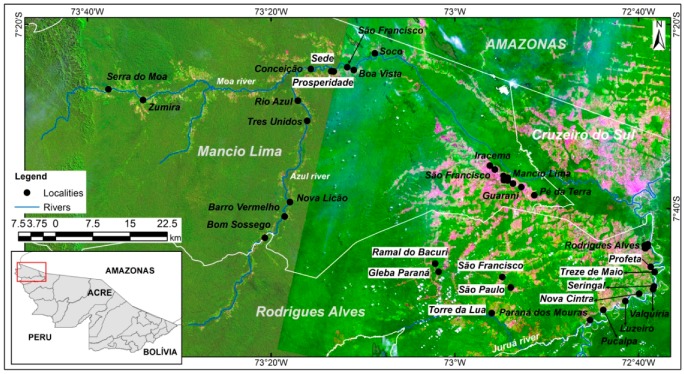
Malaria hotspot in northwest Acre, Brazil. The dots indicate the localities included in the study.

The study area comprises the municipalities of Mâncio Lima (ML) and Rodrigues Alves (RA) located in the northwest Acre state, Brazil (Figure 1). ML and RA belong to the Alto Juruá region, where currently 90% of Acre’s malaria cases are concentrated [23]. The last demographic census [30] counted 15,206 inhabitants in ML, of which 8750 live in urban areas (57.5%), and 14,389 inhabitants in RA, with 4315 residents in urban areas (29.98%). Agriculture and cattle raising are the primary economic activity of the two municipalities, making up more than 55% of the Gross Domestic Product (GDP) [31].

Malaria in Brazil is mainly caused by *Plasmodium vivax* and *Plasmodium falciparum,* transmitted by mosquitoes of the genus *Anopheles*. Since the 1980s, falciparum malaria has decreased both in transmission and mortality, being considered close to eliminated in some areas. Vivax malaria currently accounts for more than 80% of the malaria burden in Amazonia [32]. In Acre, 14,000 cases of *P. vivax* and 4944 cases of *P. falciparum* were reported in 2015 [33]. The state is still considered at high risk for *P. falciparum,* which represents around 30% of the total cases, well above the Amazon average of less than 10% [33]. In the Alto Juruá, malaria cases are found in urban areas, in riverine areas and rural settlements. Urban malaria has been attributed to the persistence of favorable *Anopheles* breeding conditions such as flooding plains and fish farming activities [24] and also to the movement of people between riverine, rural and urban areas [21]. 

The geographical location of the communities is associated with different historical contexts, as a result of past and present processes of regional space production. The riverine occupation of the Moa and Azul Rivers dates back to the period of rubber exploitation during the late nineteenth to early twentieth century. The roadside localities within the municipality of RA have their origin, in general, in the implementation of settlement projects by the National Institute of Colonization and Agrarian Reform (INCRA), post 1970s. The latter is characterized by small and medium-sized rural properties distributed along secondary roads perpendicular to the main road. This process of colonization at the fringe of the forest has placed less immune populations at the most vulnerable spots at the end of roads, where they work clearing the forest, creating habitats for the malaria vector [20].

### 2.2. Data 

Data for this study came from two databases, one provided by the TerraClass Project [34] and the other by the epidemiological study described in Lana et al. [19]. The TerraClass Project database (Available in http://www.inpe.br/cra/projetos_pesquisas/dados_terraclass.php) provided land use and land cover data for the study area. This Project aims to produce land use and land cover maps over areas that have been classified as deforested in the Legal Amazon. Currently, there are five maps produced for 2004, 2008, 2010, 2012 and 2014. These maps establish 12 classes of land use and land cover for the Brazilian Amazônia region: deforestation, urban areas, mining, mosaic of uses, annual crops, herbaceous pasture, shrubby pasture, pasture with bare soil, regeneration with pasture, secondary vegetation, others, and non-observed areas. The classification methodology and a more detailed discussion of these 12 proposed classes are found in Almeida et al. [35].

Lana et al. [19] created a dataset from a household survey (n = 520) carried out in 2015 in 40 localities within the Mâncio Lima and Rodrigues Alves municipalities (Figure 1). The survey investigated the contribution of household and locality level traits on the prevalence of malaria. Locality is the territorial unit used by the local malaria surveillance teams, and it is officially classified as urban or rural [30]. Rural localities are further divided into: indigenous villages, colonies, farms, settlements, and od rubber plantations. In urban localities (neighborhoods), sampling was systematic and probabilistic. However, in rural localities, in particular, riverine localities, there was no sampling frame, since information about most localities and their population size were unknown or outdated at the time the survey was carried out. For these localities, information was gathered based on a key informant methodology because in these places, very small variability was detected in the educational profile and the way of life of the residents. Householders, 18 years of age or more, who could answer for the rest of the household, answered the questionnaire. This survey provided the variables required for the construction of urbanization intensity indicators and are related to the presence of goods in the household, occupation and income of the householders, water supply, garbage disposal and the source of electricity in the locality, as detailed in Table 1. Variables were simplified or aggregated at locality level for the construction of indicators.

### 2.3. Urbanization Model 

We used the urban representation model proposed by Dal’Asta et al. [25] to derive an urbanization gradient for the study area. Based on the concept of extensive urbanization [26], the model postulates that the urbanization process in the Amazon can be represented by the characterization of its spatial forms, its social content and their temporal evolution.

Spatial forms constitute population settlements of various densities, cities, towns and localities and the forms created or transformed by technology, such as the construction of a dam or the mechanization of agricultural areas. These forms are the objects in the representational model, and they are part of a system of objects, that in the spatial perspective correspond to the arrangement of land uses present in the observed landscape. 

The social content corresponds to the set of values disseminated by the cities: consumption patterns and elements of “living” in the cities establish possibilities to describe this dimension [25]. This social content must be adapted to the peculiarities of each region to capture the population behavior and the city’s influence over the territories with which it connects. It captures the degree of urbanicity involved in urban-rural, or city-countryside linkages which are often y invisible and is part of a system of values.

Both, the spatial forms and the social content dimensions are associated with intensity levels that indicate stages of urbanization, thus establishing a gradient of urbanization. Changes in these intensities reflect human action, or the actors and processes that establish the rhythms or evolution of urbanization that comes from consolidated urban areas and extends in the form of urbanicity indicators, to other areas whether they are originally rural or urban. These intensity changes in both objects and values are linked with human actions, and from a representational perspective, they are part of a system of actions.

Thus, these three set of elements—objects, values and actions—contain the elements for the representation of the urbanization processes as a gradient of urban intensities in the Amazonian space, detailed in Dal’Asta et al. [25], and explored in this study. To operationalize an urban gradient with these three dimensions—objects, values and actions, we need to define:

1. A local reference: intensity levels are defined in relation to a local reference that establishes what is the most urban setting in the analyzed context. Intensities vary from the highest urban grade at the local reference to the lowest urban grade at the places less similar to the local urban grade reference. For this study, the town of Mâncio Lima (ML) was adopted as the local urban reference because it has the largest number of inhabitants and is considered a place of greater economic and administrative importance by the population, when compared to RA, which was also inventoried.
2. Spatial unit of analysis and data integration: each of the 40 localities inventoried by Lana et al. [19] corresponds to the spatial observation units.
3. Set of variables: a minimum set of variables is required so that urbanization intensity indicators can represent the complexities of the Amazonian urbanization process. This set of variables is used as proxies of each dimension analyzed, and they were selected based on Dal’Asta et al. [25] criteria. The lack of longitudinal household survey data precluded the analysis of the evolution of urbanization, as originally proposed for the action dimension.

#### 2.3.1. Characterization of the Spatial Forms: System of Objects 

Data from the TerraClass Project [34] for 2014 were used to characterize the system of objects. For each locality, a 500 m buffer was drawn to characterize the spatial context of the localities. This radius corresponds to the shortest distance between two localities in the study site. The proportion of land use and land cover classes (class composition within a buffer area) defined the position of each locality along the spatial form gradient. From TerraClass, “urban area” refers to places where the population is concentrated, and the density of streets, houses and buildings is identifiable in medium resolution images [35]. Here, the “urban area” was subdivided into: “city” referring to the municipal base of ML; “town” for the municipal base of RA and “space units of human occupation—SUHO” (TerraClass “mining” class was here aggregated to the “SUHO” class because it represents nodes of the urban fabric that articulate the local to an urban-industrial developing mode.) referring to other areas of population concentration mapped by TerraClass. 

**Figure 2:**
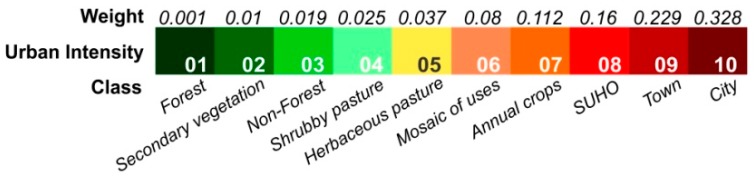
Land use and land cover class gradient associated with urban intensity. Weights assigned to each class are described in the text.

While the classes, “city”, “town” and “SUHO” represent socio-spatial forms directly associated to urbanization, the remaining classes are ordered according to the use of modern techniques, indicative of natural cover transformation into urban expressions. Details on the development of this gradient composition are found in Dal’Asta et al. [25]. Adapting the TerraClass class legend, “forest” represents the lowest intensity level of the urban phenomenon while “city”, represented by the MLbase, represents the highest urban intensity. Intermediate classes (Figure 2) according to increasing urban intensity are: “secondary vegetation”, “non-forest”, “shrubby pasture” (TerraClass “shrubby pasture” and “regeneration with pasture” classes were aggregated as a “shrubby pasture” class.), “herbaceous pasture” (TerraClass “herbaceous pasture” and “pasture with bare soil” classes were aggregated as the “herbaceous pasture” class.), “mosaic of uses”, “annual crops”, “SUHO” and “town”.

**Figure 3:**
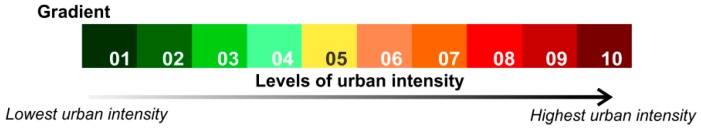
Gradient of urban intensities associated with the system of objects.

Following the methodology defined by Dal’Asta [36], a weight value reflecting urban intensity was associated to each class of land use and cover (Figure 2). These weight values were obtained from pairwise comparison method, developed by Saaty [37] for the Analytic Hierarchy Process (AHP), based on the relative importance of each class for the urbanization process (Figure 2). Classes from 06 to 10 received higher weight values because they more directly represent spaces of reproduction of urban life, while classes from 01 to 05 are classes of land cover, from which land use is inferred, representing objects of lower intensities in the gradient. The “Forest” class received the value of 0.001, which although low, is a value different from zero, due to the possibility of use, for example by extractivism. For every locality buffer (objects), the percentage of each land use and cover class was multiplied by its respective weight and then summed in order to obtain an urban intensity value per object. Thus, each locality can be positioned in a simplified gradient represented by 10 reference points (levels) of urban intensity (Figure 3) that establish a relation with the reference system (Figure 2).

#### 2.3.2. Characterization of the Social Content: System of Values

A set of 15 indicators from the household variables aggregated at the locality level collected by Lana et al. [19] survey was proposed to build the system of values. The indicators characterize the individual consumption, collective consumption, and way of life in each locality that relates to urban values as explained in Table 2 and were constructed based on the set of variables listed in Table 1.

Based on the premise that the urban phenomenon should be contextualized in a given geographic space, the system of values intensity gradient was calibrated using the values observed in the five localities classified as “city”, all of them are located within the local urban reference, the city of ML. Thus, for each indicator in Table 2, a distance value, based on the Environmental Performance Index (EPI) [40], was calculated. This method computes the distance between a locality *i* and the reference urban locality (ML) regarding the indicator *j*. The distance *D_ij_* is a dimensionless measure that varies from zero, indicating a locality that differs the most from the urban reference (ML), to 1, representing a locality that shares the same values with the local urban reference, and was calculated using Equation (1):

(1) $$D_{ij}=\frac{\left({\mathrm{ML}}_{j}-\min_{j}\right)-\left({\mathrm{ML}}_{j}-P_{ij}\right)}{\left({\mathrm{ML}}_{j}-\min_{j}\right)},1\leq i\leq 35\;\mathrm{and}\;1\leq j\leq 15$$

where $D_{ij}$ is the distance of a locality *i* to in relation to ML city for the indicator *j*. ${\mathrm{ML}}_{j}$ is the value of indicator *j* in the ML city. $\min_{j}$ is the lowest value observed for indicator *j* in the studied area. $P_{ij}$ is the value (proportion) of the indicator *j* in the locality *i*.

A Synthetic Index (SI) was constructed to form an urban intensity gradient for the system of values. In this gradient, the urban intensities are represented on a scale of 0 to 1, and the closer to 1 the more “similar” the place is to the behavior observed in ML. To compute the SI, the distances of each indicator were summed and the result linearly normalized (Equation (2)):

(2) $$SI_{i}=\overset{15}{\underset{j=1}{\sum }}D_{ij},1\leq i\leq 35$$

where: $SI_{x}$ is the Index Synthetic of a locality *i* in the system of values.

## 3. Results

**Figure 4:**
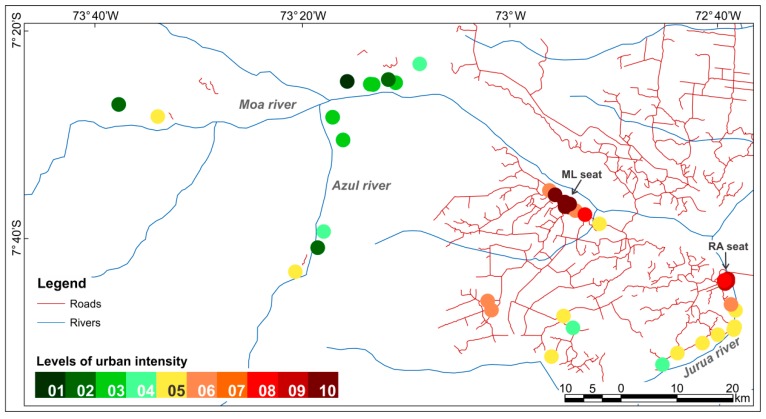
Urban intensity gradient associated with the system of objects in northwest Acre.

In general, the results reveal an urban-rural gradient in the study area from the municipal seats, passing through the localities along the road networks and ending at the riverine localities. Figure 4 shows the classification of the localities according to their spatial forms (system of objects). In Figure 4, the local urban reference, the ML seat, is seen as part of a cluster of localities classified from high to intermediate intensity (levels 5 to 10). There is a secondary urban cluster centered at the RA seat (longitude 72°40′ W) that extends southward along the Juruá River, where there are five localities classified at intermediate levels of intensity (levels 4 and 5). There is a set of five localities accessible by dirty roads also classified as intermediate levels of intensity (levels 4 to 6). At the left side of the map, one finds the less urbanized localities, a set of 13 localities only accessible by river, which were classified in the lower levels of intensity (levels up to 4).

The distribution of spatial forms of these localities reflects different actors and processes that historically produced the regional space, from the most recently created settlements accessed by the road networks connecting them to the nearest city (RA); to the oldest areas of occupation along the rivers that are smaller in population. Rural settlements accessible by road are located in landscapes dominated by land uses associated with pastures (45.94%), “mosaic of uses” (21.07%) and “forest” (16.25%), while riverine localities are predominantly associated with classes of “forest” (48%), “secondary vegetation” (20%), “pasture” (18.13%) and “mosaic of uses” (8%).Riverine localities represent the less impacted areas, associated with the traditional occupation of the Amazon.

**Figure 5:**
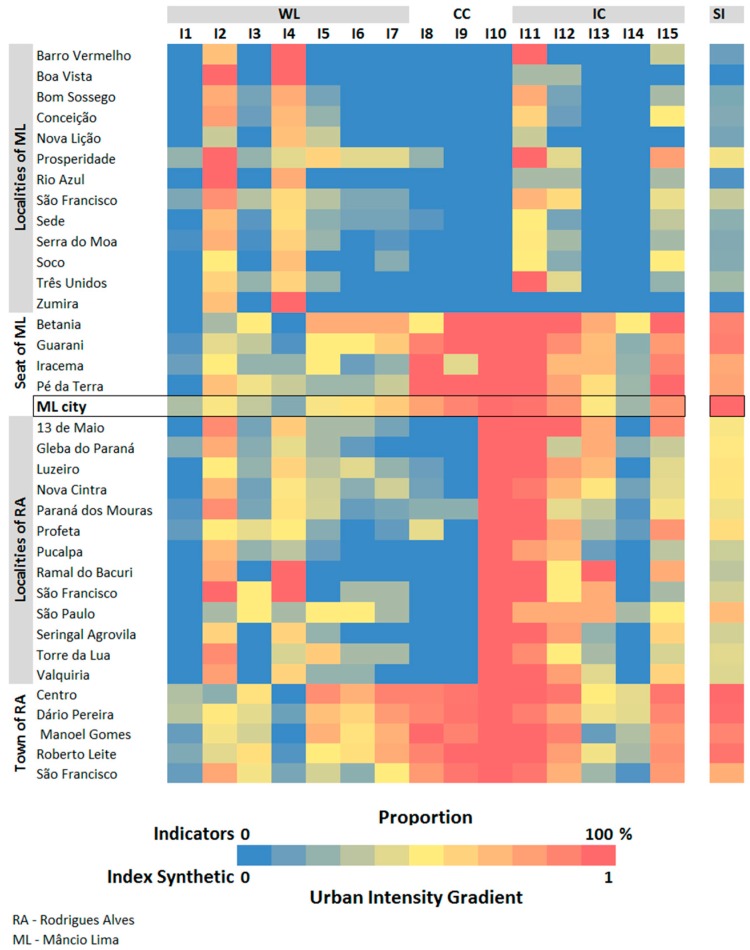
Composition of the indicators and the Synthetic Index (SI) of the system of values for localities in northwest Acre. The indicators reflect the proportion of households in the locality equipped with: social networks (I1); social benefits (I2); Internet access (I3); income exclusive from “rural” activities (I4); at least one “urban” activity income (I5); indoor shower (I6); indoor place for needs (septic tank or toilet) (I7); network water supply (I8); regular garbage collection service (I9); electricity from the public network (I10); TV (I11); blender (I12); motorcycles (I13); computers (I14); cellular devices (I15). WL: related to Way of Life; CC: Collective Consumption; IC: Individual Consumption.

Figure 5 shows the values of the individual indicators that composed the system of values’ Synthetic Index (SI) for each locality. Households at the riverine localities lack collective consumption goods (network supply water, regular garbage collection service, and electricity from the public network), as well as computers and motorcycles. On the other hand, cellular devices (present in 10 localities), internet (present in 7 localities), blenders (present in 10 localities) and television (present in 12 localities) are part of the everyday life of this riverine population. In these localities, power grid electricity is absent, and energy is supplied by generators. The absence of an enclosed room for bath and other needs, which is a typical of traditional Amazonian living, was detected in most riverine localities (70%). In turn, in the road-accessible localities, an enclosed room for bathing was absent in only four localities (28%) while a room for needs was absent in five localities (36%). Moreover, garbage collection (one locality), water supply (four localities), computers (in five locations and covering 4.5–25% of households), internet access (nine localities and up to 50% of households) and electricity supply is available in all localities. At least 25% of households have mobile phones. Televisions and motorcycles are present in all localities with proportions higher than 75% and 10% of all households, respectively.

A blender is a non-essential consumption good usually found in households serviced with power grid energy, being more frequent in the municipal seats. Blenders are present in all road-accessible localities, with proportions varying from 33.3% to 100%. In 10 riverine localities (77%), the proportions of households with a blender varied from 12.5% to 57%. Access to social networks and the presence of computers is strongly associated with households in municipal seats: the proportion of households in these localities with computers vary from 4% to 40.9% and access to social networks is up to 53%. In riverine localities and roadside localities, only six (22%) have households with access to social networks and five (18%), all located in RA, have computers.

In general, the composition of household income stems from both traditional rural activities and urban activities, regardless of the household’s location. In only five localities, household income is derived exclusively from rural activities, whereas, in three neighborhoods (Centro and Manoel Gomes (RA), and Betânia (ML)) income is associated only with urban activities. In other words, the indicators, “households in which income is exclusive from rural activities” and “households in which at least one of the income generating activities comes from urban activities” show that, even in the municipal seat, there are households whose source of income originates from rural activities. Meanwhile, households located in the interior of the municipality have at least part of their sources of income in urban activities. In only seven localities (17.5%), urban activities do not contribute to the household income. The proportion of households with at least one urban activity making up their income ranges from 22.2% to 86.3% in the municipal seats and in the other localities, reaches up to 62.5%. On the other hand, households with income derived exclusively from rural activities account for more than 40% and 25% of the households surveyed in the riverine localities and roadside localities, respectively, and up to 33% are in the municipal seat of RA. 

**Figure 6:**
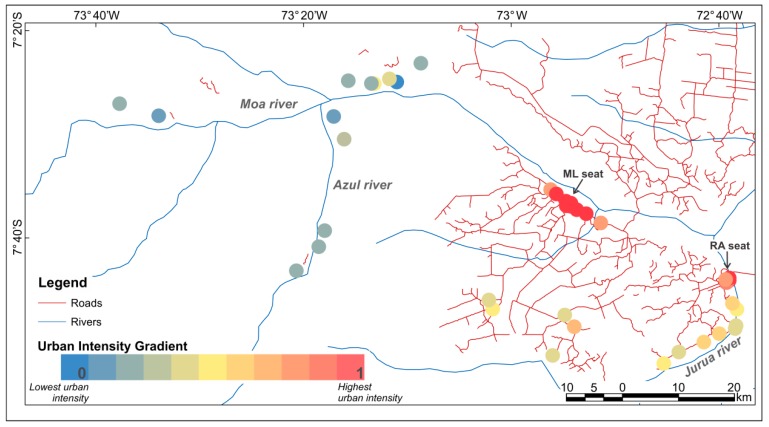
Gradient of intensities of *system of values*’ Synthetic Index (SI) in northwest Acre.

The system of values resulted from the integration of individual indicators, summarized by the Synthetic Index (SI). From the SI spatial distribution (Figure 6), the less urbanized places are the 11 riverine localities in Moa and Azul Rivers, classified with SI values up to 0.24. In this region, only two localities presented intermediate levels of urbanization. A second cluster of intermediate positions in the gradient (SI values of 0.3–0.68) comprises 12 localities: five of them accessible by dirt roads and seven along the Juruá River. A third cluster, centered on the ML seat, represents localities with SI values above 0.76. The neighborhoods of the RA municipal seat represent another cluster of localities with higher levels of urbanization.

**Figure 7:**
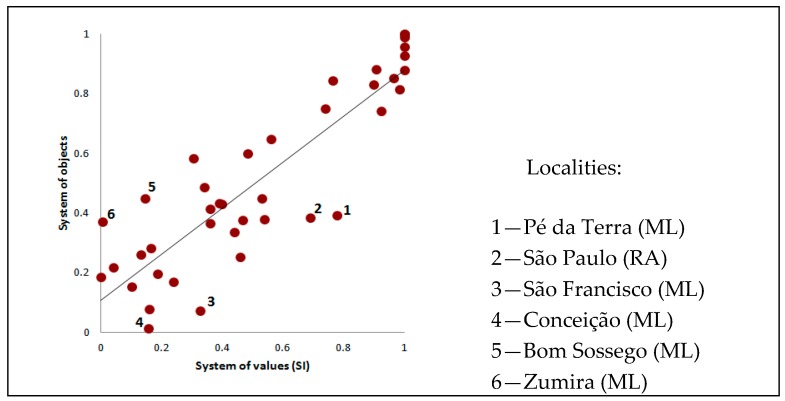
Scatterplot of value and object systems. The two indicators are highly correlated (Pearson’s r = 0.88, 95% confidence interval = [0.79,0.93]).

Exploring the relation between objects of urban intensity and SI values (Figure 7) one can observe that in general, the system of objects and the system of values are directly correlated (Pearson’s r = 0.88). Most localities present similar positions along the gradients. Exceptional localities are highlighted (numbered) in Figure 7, and they refer to places with urban intensities (high, intermediate or low) differentiated between the system of objects and values. These localities occur preferentially within the ML municipality.

**Figure 8:**
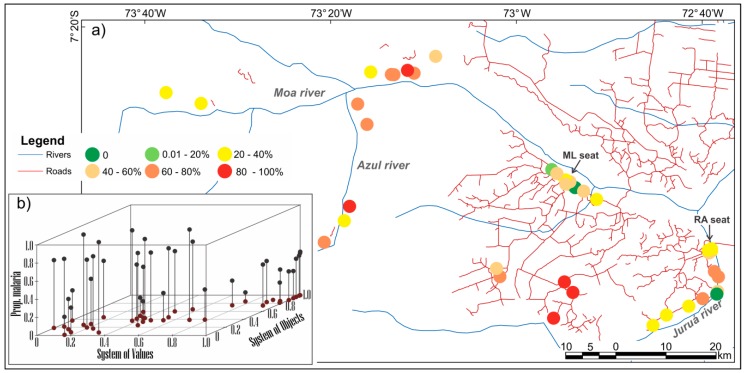
(**a**) Distribution of percentages of malaria-positive households (at least one resident with malaria in the last 12 months). In detail (**b**) system of values Synthetic Index (x), system of objects (y) and the percentage of households with malaria (z) for each locality.

From these results, the urban phenomenon and its values are shown to be widespread in the territory, along a gradient. Similarly, malaria occurrence also reveals a complex spatial distribution with respect to its exposure and risks. Along with this urban gradient, malaria occurs with different intensities. The distribution of percentages of malaria-positive households (defined as at least one resident with malaria in the last 12 months) by locality in the year of the household survey, is shown in Figure 8a. Malaria occurs independently of the rural or urban situation of the households, although there are more positive households in the road settlements and riverine localities. Figure 8b shows the behavior of each locality in the system of object and values in relation to malaria. According to Figure 8b, there is an increased trend of positive households in places with low to medium urban intensities. In addition, localities with high urban intensities present low to intermediate patterns of malaria occurrence and intermediate levels of malaria occurrence are also associated with intermediate urban intensities. In other words, there are complex patterns of association between the occurrence of malaria and the urbanization of the territory in the study area which are imperceptible in dichotomous representations.

## 4. Discussion

The effects of urbanization on the risk of malaria and transmission of *Plasmodium* are still poorly understood and it is not clear whether urbanization will transform malaria from a rural disease to an urban disease [10]. Imprecision in defining what characterizes urban spaces, what urban malaria is, and whether malarial control should differ between rural and urban areas, have an impact on the analysis of available data and the development of intervention strategies [10]. 

Given this context, the fragility of the political-administrative definition for the determination of rural and urban spaces in regions where malaria is endemic is shown in this paper for the northwest Acre. Although the units of analysis adopted in this study represent urban centralities on a basal scale [41] and are officially classified as “rural”, they differ from each other and present characteristics that range from traditional Amazonian behavior, to patterns of consumption typical of industrial urban society. The Malaria Elimination Plan in Brazil [42] states that malaria control should be planned and organized at the municipal level. The plan starts with the definition of the priority localities, which are indigenous areas, mining sites, recent rural settlements and riverine populations. According to our results, this classification represents different levels of integration and distancing between “rural” and “urban” spaces. The recognition of an urban gradient constitutes the basis for directing resources to neglected and preferential populations [7]. 

Spatial representations based on a gradient of intensities recognize that dichotomous categories are insufficient for characterizing the diversity of the spaces of daily life in the Amazon. Our analysis corroborates the findings of Lana et al. [19], who have already pointed to a gradient, based on the socioeconomic characteristics of the households within this study area. Lana et al. [19] classified the localities into three groups: municipal seats (combining ML and RA), RA rural localities and ML rural localities. Our results broadened this gradient and provide a classification, defined in levels of urban intensity, driven by the malaria data and not by an arbitrary definition of urban/rural. There is an ecological diversity in the exposure patterns and prevention opportunities in the Amazon context that is not considered when adopting dichotomous categories to explain malaria risk (urban vs. rural risk)—a point that is fundamental for malaria intervention strategies [10].

The ambiguity in defining the urban space makes it difficult to determine whether a case is urban malaria or not, a priority issue for defining control strategies [10]. This also compromises understanding the risk of exposure to disease and its prevention. The mobility and circulation of *Plasmodium* are affected by particular characteristics of the study site. Some urban spaces present typical “rural” features, like the presence of fishponds in the cities [22,24]. Also, as shown by the indicators associated with the composition of income discussed in this study, there was a high number of the urban population whose income is exclusively from “rural” activities. Another problem is related to disease notification. Often, parasitic malaria infections acquired outside urban areas are assigned as urban transmission because they are diagnosed in cities where health facilities are more accessible [10]. This fact, in addition to uncertainties about locality rural/urban classification, commits to understanding urban malaria. Improving local knowledge across the Amazon region on the relative contribution of vectors, parasites, humans and the environment to the persistence of *Plasmodium* transmission could facilitate the elimination of malaria, especially because the underlying factors may not be the same throughout the region [22]. 

In complex landscapes such as the Brazilian Amazon, the urban phenomenon should be contextualized considering the inherent characteristics of the different locations [43,44]. This approach corresponds with the perspective of the United Nations Human Settlements Program (UN-HABITAT), because it is necessary to consider the patterns and references of the regional space in order to discuss urban and territorial plans in all of their dimensions [45]. By shifting the urban reference, local patterns and behaviors that are obscured by the national metrics, such as those used by the Brazilian Institute of Geography and Statistics (IBGE) [46] emerge, surpassing the Amazonian discourse as a rural region and a demographic void [47]. Although not densely populated, the arrangement of localities in northwest Acre shows the contribution of both traditional and driven occupation centers, as expressed by the term urbanized forest [29], corroborating Amaral et al. [41], Parry et al. [48] and Guedes et al. [49] in their characterization of the Amazonian rural space. 

In the Alto Juruá region, the municipalization of malaria control was initiated in 2014. Previously funded and carried out by agents from the state level, the transference of control to poor municipalities resulted in a decrease in the frequency of active searches for malaria cases, and the deactivation of many health posts that used to assist the population that does not have the financial resources to seek care in the city. This situation has consequences for the elimination of malaria. The main issue is effectiveness of the search for active cases, which in 2014 represented 16% of the confirmed malaria cases for the Amazon Basin [22]. This control strategy, adopted by the Brazilian Ministry of Health since 1996, seeks to identify cases of asymptomatic malaria, which, if left untreated, continues to transmit the infection. This strategy is essential for interrupting malaria transmission [50,51]. In regions where malaria is endemic, and the population has acquired some immunity, the percentage of asymptomatic cases is high [51,52]. Asymptomatic individuals may represent a significant malaria reservoir, not only in communities isolated by rivers but also in densely populated mining villages [52]. The most important operational difference between a control and an elimination program is the concentration of activities to identify and attack the clinical and asymptomatic infections that perpetuate transmission [50]. 

In the Amazon, the type of access (roads or rivers) define the connection and flow of a place and reveal the different rhythms of its transformation. The system of object characterization showed riverine and roadside localities embedded in differentiated land use mosaics. For riverine localities, less impacted landscape predominated, while for roadside localities, pastoral coverage dominate the landscape. In addition, in the descending water phase, the localities of Serra do Moa and Bom Sossego (further upstream of ML) are several days away by boat from the f ML seat. To those settlements connected to city by roads, the same distance is traveled in hours, which is why motorbikes are favored by the local inhabitants and can be found in 100% of the households in some areas. With respect to malaria, localities in different landscapes have different exposure and connectivity hence they require different actions for malaria control. 

The change and/or incorporation of new habits is one of the challenges for malaria elimination since the perception of individual risk can affect the acceptance of interventions and hinder elimination objectives [22]. By connecting different places in the territory and disseminating urban behaviors, extensive urbanization can act as a way to sensitize individuals and communities to maintain or adopt new behaviors, reducing the infection risk. Individual consumer goods could be useful for new campaigns to promote behavior changes. From our results, these goods are widely present in households regardless of its location. Furthermore, according to the survey on the use of Information and Communication Technologies (ICT) in Brazilian households [53], expansion in the presence and use of these goods is expected. Internet access in the national territory, for example, increased from 39% to 61% between 2008 and 2016 [53].

Our results also showed that even the most isolated localities, with limited access to urban centers and distant from the main axes of circulation of goods and information, articulate urban behaviors. Therefore, we cannot categorize them as strictly “rural”, either from income-generating activities, from traditional production models, or patterns of consumption and access to goods and services of universal use. Contemporary rural areas can only be understood as a continuum of urban intensities, from a spatial perspective. Also, based on the organization of economic activity, cities can no longer be identified only with industrial activity, nor can the countryside be only identified with agriculture and cattle raising [54].

## 5. Conclusions

In this work, we discuss the rural-urban classification dichotomy and consider its importance for malaria surveillance in northwest Acre, based on indicators for 40 localities in the municipalities of ML and RA. The use of an urbanization model [25] allowed us to characterize the landscape gradients associated with malaria risk first identified by Lana et al. [19] for these localities. In general, the systems characterization captured the extension of urbanization beyond the municipal seats, showing that the urban phenomenon reaches places in the territory with different intensities, elucidating the adherence of the extensive urbanization thesis to this part of the Amazon. Along this urban gradient, malaria occurs with different intensities, although there is an increased trend in positive households in the areas of road settlements and riverine localities, places with low to medium urban intensities.

Our results show that localities, officially classified as rural, are inserted in contexts that are not essentially urban or rural, both from their socio-spatial forms and content, corroborating the argument that the closer relationship between populations of the countryside with urban centers is an observable phenomenon throughout the Amazon. Our results also contribute to Lana et al. [19] to better understand the local characteristics of malaria-endemic areas. The malaria versus urbanization patterns that we found may be specific for this study area where malaria is highly endemic. Other studies should investigate whether this pattern stands in other regions. Moreover, this methodology may also be useful for discussing other diseases with patterns influenced by urbanization, that also demand non-arbitrary classifications. However, as the model has a strong empirical basis, it must be adapted to the local context, as well, the data used to characterize the dimensions of the model must consider the inherent limitations of the model itself. Datasets, such as the one by Lana et al. [19], are essential to capture relationships and processes of interest to epidemiological surveillance on the local scale often invisible in secondary and aggregate data, such as the census tracts. From this, considering the position of each locality in this urbanization gradient, one can move forward in the discussion of normative definitions of “urban” and “rural” for public health policies and actions to control and eliminate malaria. It is worth noting that although *Anopheles darlingi*, the main mosquito vector of *Plasmodium* in Brazil, is present all over the country, the incidence of malaria is almost exclusively restricted to the Amazon region, where a series of biological, geographical, ecological and social factors favor the transmission of the disease and impair the use of standard control procedures [55].

## Figures and Tables

**Table 1:** Household traits from Lana et al. [19] data used as variables for urbanization intensity indicators.

Group ^1^	Information	Variables
Household goods	Ownership of goods	TV; blender; motorcycle; computer; Facebook account; internet on smartphone or computer.
Occupation and income	Income by householders’ occupation type	Income derived from: agriculture, cattle, fishing, subsistence agriculture surplus, forest products, crafts and domestic labor. Income derived from: public service, cleaner, work with a formal contract, driver or boatman, social benefits (income transfer programs), general services (school positions, bus ticket collector).
Water supply	Water access Bathroom facilities	Piped water within the residence or only in the yard. Shower facility indoors and enclosed place for needs.
Garbage disposal	Waste destination	Household garbage collected by the public service.
Access to electricity	Electricity inside household	Electric power supplied by conventional network.

^1^ According to the household survey structure [19].

**Table 2:** Indicators used to characterize the system of values, divided into (Group): related to Way of Life (WL); Collective Consumption (CC); Individual Consumption (IC).

Group	Indicators	Justification
WL	I1—Proportion of households with a Facebook account	These indicators refer to new arrangements in the countryside regarding the occupational profiles of its population and the introduction of elements of rationality and modernity disseminated by the cities. This mediation seeks to understand the contemporary transformation of the official rural settlements considering a context of extensive urbanization [26]. The indicators associated with the type of bathrooms (I6 and I7) refer to the traditional behavior of the Amazonian people and the insertion of new habits. The presence of social benefits, in some cases associated with traditional activities, indicate trade monetization and insertion of the population into a market economy.
	I2—Proportion of households receiving social benefits (Bolsa Família, Bolsa Verde e Bolsa Pesca) ^1^	
	I3—Proportion of households with internet access	
	I4—Proportion of households in which income is exclusively from “rural” activities	
	I5—Proportion of households in which at least one source of income is from “urban” activities	
	I6—Proportion of households with shower indoors	
	I7—Proportion of households with an enclosed place for needs (septic tank or toilet)	
CC	I8—Proportion of households served by water supply system	These indicators refer to the numerous goods and services (of universal value) that tend to be produced and consumed at a collective level in the cities [38]. The expectation of access to these services offered preferentially in the cities, reveals the city influence is extrapolating its physical dimension [39].
	I9—Proportion of households with regular garbage collection service	
	I10—Proportion of households linked to power grid electricity network	
IC	I11—Proportion of households with TV	These indicators refer to the social process of appropriation of consumer goods by individuals. In the context of this work, they are equipment whose access is facilitated and diffused in cities.
	I12—Proportion of households with blender	
	I13—Proportion of households with motorcycle	
	I14—Proportion of households with computer	
	I15—Proportion of households with cellular phone	

Note: ^1^ Bolsa Família: federal government income transfer program directed at families in poverty (monthly income between R$ 85.01 (U.S.$26.20) and R$ 170.00 (U.S.$52.31) per capita) and extreme poverty (monthly income up to R$ 85.00 (U.S.$26.20) per capita, provided to mothers that are pregnant or that have children between 0 and 17 years old) throughout the country. This benefit, seeks to guarantee these families the right to food and access to education and health. Bolsa Verde: federal income transfer program for families in extreme poverty conditions, living in areas of relevance to environmental conservation. It works as an incentive to communities to keep using their land in a sustainable way. The program, created under the “Brasil Sem Miséria” program, grants R$ 300 (U.S.$92.31), every three months, to beneficiary families for two years, and can be renewed. Bolsa Pesca or Seguro Defeso: federal government program grant aid of four months minimum wage (U.S.$ 293.54) to artisanal fishermen during the breeding season when fishing is prohibited. The current exchange rate on20 February, 2018 was: U.S.$1 to R$ 3.25.

## References

[1] Arnaiz-Schmitz C., Schmitz M.F., Herrero-Jauregui C., Gutierrez-Angonese J., Pineda F.D., Montes C. (2018). Identifying socio-ecological networks in rural-urban gradients: Diagnosis of a changing cultural landscape. Sci. Total Environ..

[2] Limonad E., Monte-Mór R. (2015). Beyond the right to the city: Between the rural and the urban. Rev. Electrón. Geogr. Cienc. Soc..

[3] Nagendra H., Unnikrishnan H., Sen S. (2014). Villages in the city: Spatial and temporal heterogeneity in rurality and urbanity in Bangalore, India. Land.

[4] Taccoli C. (2003). The links between urban and rural development. Environ. Urban..

[5] Lourenço A.E.P. (2012). The meaning of ‘rural’ in rural health: A review and case study from Brazil. Glob. Public Health.

[6] O’Reilly G., O’Reilly D., Rosato M., Connolly S. (2007). Urban and rural variations in morbidity and mortality in Northern Ireland. BMC Public Health.

[7] Hart L.G., Larson E.H., Lishner D.M. (2005). Rural definitions for health policy and research. Am. J. Public Health.

[8] Cyril S., Oldroyd J.C., Renzaho A. (2013). Urbanisation, urbanicity, and health: A systematic review of the reliability and validity of urbanicity scales. BMC Public Health.

[9] Jones A.D., Acharya Y., Galway L.P. (2016). Urbanicity gradients are associated with the household- and individual-level double burden of malnutrition in sub-Saharan Africa. J. Nutr..

[10] Wilson M.L., Krogstad D.J., Arinaitwe E., Arevalo-Herrera M., Chery L., Ferreira M.U., Ndiaye D., Mathanga D.P., Eapen A. (2015). Urban malaria: Understanding its epidemiology, ecology, and transmission across seven diverse icemr network sites. Am. J. Trop. Med. Hyg..

[11] Hay S.I., Guerra C.A., Tatem A.J., Atkinson P.M., Snow R.W. (2005). Urbanization, malaria transmission and disease burden in Africa. Nat. Rev. Microbiol..

[12] Hall S.A., Kaufman J.S., Ricketts T.C. (2006). Defining urban and rural areas in U.S. Epidemiologic studies. J. Urban Health.

[13] Siri J.G., Lindblade K.A., Rosen D.H., Onyango B., Vulule J., Slutsker L., Wilson M.L. (2008). Quantitative urban classification for malaria epidemiology in sub-Saharan Africa. Malar. J..

[14] Cohen S.A., Cook S.K., Kelley L., Foutz J.D., Sando T.A. (2017). A closer look at rural-urban health disparities: Associations between obesity and rurality vary by geospatial and sociodemographic factors. J. Rural Health.

[15] Helbich M., Blüml V., de Jong T., Plener P.L., Kwan M.-P., Kapusta N.D. (2017). Urban–rural inequalities in suicide mortality: A comparison of urbanicity indicators. Int. J. Health Geogr..

[16] Sacks J.D., Rappold A.G., Davis J.A., Richardson D.B., Waller A.E., Luben T.J. (2014). Influence of urbanicity and county characteristics on the association between ozone and asthma emergency department visits in North Carolina. Environ. Health Perspect..

[17] Novak N.L., Allender S., Scarborough P., West D. (2012). The development and validation of an urbanicity scale in a multi-country study. BMC Public Health.

[18] Tatem A.J., Gething P.W., Smith D.L., Hay S.I. (2013). Urbanization and the global malaria recession. Malar. J..

[19] Lana R.M., Riback T.I.S., Lima T.F.M., da Silva-Nunes M., Cruz O.G., Oliveira F.G.S., Moresco G.G., Honório N.A., Codeço C.T. (2017). Socioeconomic and demographic characterization of an endemic malaria region in Brazil by multiple correspondence analysis. Malar. J..

[20] Da Silva-Nunes M., Moreno M., Conn J.E., Gamboa D., Abeles S., Vinetz J.M., Ferreira M.U. (2012). Amazonian malaria: Asymptomatic human reservoirs, diagnostic challenges, environmentally driven changes in mosquito vector populations, and the mandate for sustainable control strategies. Acta Trop..

[21] Angelo J.R., Katsuragawa T.H., Sabroza P.C., de Carvalho L.A.S., de Silva L.H.P., Nobre C.A. (2017). The role of spatial mobility in malaria transmission in the Brazilian Amazon: The case of Porto Velho municipality, Rondônia, Brazil (2010–2012). PLoS ONE.

[22] Ferreira M.U., Castro M.C. (2016). Challenges for malaria elimination in Brazil. Malar. J..

[23] PAHO (2016). Report on the Situation of Malaria in the Americas 2014.

[24] De Reis I.C., Honório N.A., de Barros F.S.M., Barcellos C., Kitron U., Camara D.C.P., Pereira G.R., Keppeler E.C., da Silva-Nunes M., Codeço C.T. (2015). Epidemic and endemic malaria transmission related to fish farming ponds in the Amazon frontier. PLoS ONE.

[25] Dal’Asta A.P., Amaral S., Monteiro A.M.V. (2017). Um modelo para a representação espaço-temporal do fenômeno urbano na Amazônia Contemporânea. Rev. Políticas Públicas Cid..

[26] Monte-Mór R.L.M. (2006). O que é o Urbano, no Mundo Contemporâneo.

[27] Becker B.K. (1978). Uma hipótese sobre a origem do fenômeno urbano numa fronteira de recursos no Brasil. Rev. Bras. Geogr..

[28] Becker B.K. (1974). A Amazônia na estrutura espacial do Brasil. Rev. Bras. Geogr..

[29] Becker B.K., Clüsener M.G., Sachs I. (1995). Undoing myths: The Amazon—An urbanized forest. Brazilian Perspectives on Sustainable Development of the Amazon Region—Man and Biosphere Series.

[30] IBGE, IBGE (2010). Censo Demográfico 2010.

[31] IBGE, SUFRAMA Produto Interno Bruto dos Municípios. https://cidades.ibge.gov.br/brasil/ac/rodrigues-alves/pesquisa/38/46996?ano=2014.

[32] Ministério da Saúde (MS) (2014). Situação Epidemiológica—Dados.

[33] Secretaria de Vigilância em Saúde (SVS)/MS (2017). Boletim Epidemiológico.

[34] Instituto Nacional de Pesquisas Espaciais (INPE), Embrapa (2014). Terraclass.

[35] De Almeida C.A., Coutinho A.C., Esquerdo J.C.D.M., Adami M., Venturieri A., Diniz C.G., Dessay N., Durieux L., Gomes A.R. (2016). High spatial resolution land use and land cover mapping of the Brazilian legal Amazon in 2008 using landsat-5/tm and modis data. Acta Amaz..

[36] Dal’Asta A.P. (2016). Representações do Fenômeno Urbano na Amazônia Contemporânea: Observações no Sudoeste Paraense. Ph.D. Thesis.

[37] Saaty T.L. (1980). The Analytic Hierarchy Process.

[38] Castells M. (1974). La Cuéstion Urbana.

[39] Cardoso A.C.D., Lima J.J.F., Cardoso A.C.D. (2006). Tipologias e padrões de ocupação urbana na Amazônia oriental: Para que e para quem?. O rural e o Urbano na Amazônia. Diferentes Olhares e Perspectivas.

[40] Hsu A., Johnson L., Lloyd A. (2013). Measuring Progress: A Practical Guide from the Developers of the Environmental Performance Index (EPI).

[41] Amaral S., Dal’Asta A.P., Brigatti N., de Pinho C.M.D., de Castro Medeiros L.C., de Andrade P.R., Pinheiro T.F., Alves P.A., Escada M.I.S., Monteiro A.M.V. (2013). Comunidades ribeirinhas como forma socioespacial de expressão urbana na Amazônia: Uma tipologia para a região do Baixo Tapajós (Pará-Brasil). Rev. Bras. Estud. Popul..

[42] MS (2016). Plano de Eliminação de Malária no Brasil.

[43] Barbieri A.F., Monte-Mór R.L.M., Bilsborrow R.E., Sherbiniin A., Rahman A., Barbieri A.F., Fotso J.C., Zhu Y. (2009). Towns in the jungle: Exploring linkages between rural-urban mobility, urbanization and development in the Amazon. Urban Population-Environment Dynamics in the Developing World: Case Studies and Lessons Learned.

[44] Becker B.K. (2013). A Urbe Amazônida: Entre a Floresta e a Cidade.

[45] UN-HABITAT (2015). International Guidelines on Urban and Territorial Planning.

[46] IBGE (2017). Classificação e Caracterização dos Espaços Rurais e Urbanos do Brasil: Uma Primeira Aproximação.

[47] Monte-Mór R.L.M. (2004). Modernities in the Jungle: Extended Urbanization in the Brazilian Amazon. Ph.D. Thesis.

[48] Parry L., Day B., Amaral S., Peres C.A. (2010). Drivers of rural exodus from Amazonian headwaters. Popul. Environ..

[49] Guedes G., Costa S., Brondízio E. (2009). Revisiting the hierarchy of urban areas in the Brazilian Amazon: A multilevel approach. Popul. Environ..

[50] Moonen B., Cohen J.M., Snow R.W., Slutsker L., Drakeley C., Smith D.L., Abeyasinghe R.R., Rodriguez M.H., Maharaj R., Tanner M. (2010). Operational strategies to achieve and maintain malaria elimination. Lancet.

[51] Macauley C. (2005). Aggressive active case detection: A malaria control strategy based on the Brazilian model. Soc. Sci. Med..

[52] Da Silva N.S., da Silva-Nunes M., Malafronte R.S., Menezes M.J., D’Arcadia R.R., Komatsu N.T., Scopel K.K., Braga E.M., Cavasini C.E., Cordeiro J.A. (2010). Epidemiology and control of frontier malaria in Brazil: Lessons from community-based studies in rural Amazonia. Trans. R. Soc. Trop. Med. Hyg..

[53] NIC, Núcleo de Informação e Coordenação do Ponto BR (2017). Pesquisa Sobre zo Uso das Tecnologias de Informação e Comunicação nos Domicílios Brasileiros: Tic Domicílios 2016.

[54] Silva J.G. (1997). O novo no rural brasileiro. Nova Econ..

[55] Tauil P., Daniel-Ribeiro C. (1998). Some aspects of epidemiology and control of Malaria in Brazil. Res. Rev. Parasitol..

